# Factors Related to the Differential Development of Inter-Professional Collaboration Abilities in Medicine and Nursing Students

**DOI:** 10.3389/fpsyg.2020.00432

**Published:** 2020-03-25

**Authors:** Nancy Berduzco-Torres, Begonia Choquenaira-Callañaupa, Pamela Medina, Luis A. Chihuantito-Abal, Sdenka Caballero, Edo Gallegos, Montserrat San-Martín, Roberto C. Delgado Bolton, Luis Vivanco

**Affiliations:** ^1^Escuela Profesional de Enfermería, Universidad Nacional San Antonio Abad del Cusco, Cusco, Peru; ^2^Facultad de Ciencias de la Salud, Universidad Andina del Cusco, Cusco, Peru; ^3^Departamento de Estadística e Investigación Operativa, Universidad de Granada, Melilla, Spain; ^4^Servicio de Medicina Nuclear, Centro de Investigación Biomédica de La Rioja (CIBIR), Logroño, Spain; ^5^Plataforma de Bioética y Educación Médica, Centro de Investigación Biomédica de La Rioja (CIBIR), Logroño, Spain; ^6^Centro Nacional de Documentación en Bioética, Fundacion Rioja Salud, Logroño, Spain; ^7^Area de Salud, Nutrición y Bioética, Fundación Universitaria Iberoamericana (FUNIBER), Barcelona, Spain

**Keywords:** inter-professional collaboration, empathy, lifelong learning, loneliness, subjective well-being, medicine students, nursing students, professionalism

## Abstract

**Introduction:**

For physicians and nurses, teamwork involves a set of communication and social skills, and specific training in interdisciplinary work in order to be able to work together cooperatively, sharing responsibilities, solving problems, and making decisions to carry out actions centered on patients’ care. Recent studies demonstrate that in the absence of targeted interdisciplinary educational programs, the development of teamwork abilities is sensitive to the influence of the dominant work environment. The purpose of this study was to characterize the role that environmental and individual factors play in the development of teamwork in environments with a dominant hierarchical work model.

**Methods:**

Questionnaires were distributed to 1,880 undergraduate students (980 medicine students and 900 nursing students) from three universities of Cusco city (Peru). The Jefferson Scale of Attitudes toward Physician–Nurse Collaboration was used as the main variable. The Jefferson Scales of Empathy and Lifelong Learning, the Social and Emotional Loneliness Scale for Adults, the Scale of Life Satisfaction, sex, discipline, age, and academic semester were used as explanatory variables. After calculating internal reliability and normality of the main measures, descriptive, comparative, and correlation analyses were performed to determine variables influencing the teamwork score.

**Results:**

A total of 1,518 (81%) surveys were returned fully completed. Adequate reliability was confirmed in all instruments. In the sample, nursing students showed greater inter-professional collaborative abilities than medicine students (*p* < 0.001). This attitudinal gap was higher in advanced semesters. A three-way ANOVA indicated differences in teamwork were associated with discipline (*p* < 0.001), sex (*p* < 0.01), and university (*p* < 0.001). However, main effects were associated only with discipline (η*_p_*^2^ = 0.14). Teamwork showed an inverse correlation with loneliness (ρ = −0.28; *p* < 0.001) and a positive correlation with empathy (ρ = + 0.49; *p* < 0.001) and lifelong learning (ρ = + 0.48; *p* < 0.001). Teamwork positively correlated with life satisfaction only in the medicine student group (ρ = + 0.15; *p* < 0.001).

**Conclusion:**

These findings bring new evidence to support the main effect that social environments, in the absence of targeted interdisciplinary educational programs, play in the development of teamwork.

## Introduction

In healthcare disciplines, especially in medicine and nursing, “professionalism refers to the set of skills and values that characterizes the essence of humanism of professionals who are in charge of patients’ healthcare” (Vivanco and Delgado-Bolton, 2015). This concept includes a systematic distribution of professional qualities and skills that compose the quintessence of their professional conduct irrespective of their geographical, social, or cultural differences. For evaluation objectives, a definition from the medical education framework proposes that professionalism is attained by becoming proficient in three essential domains: clinical and technical skills, communication and social skills, and a proper understanding of the ethical and legal structure of professional behavior (Stern, 2006). However, regarding communication and social skills, this domain is not restricted to patients and their families. It additionally refers to other healthcare professionals who are part of the team in charge of patients’ care. As stated by the World Health Organization [WHO] (2010), a positive inter-professional collaborative work, also called “teamwork,” is described as a capability of “multiple healthcare workers from different professional backgrounds to provide comprehensive services by working with patients, their families, their professional careers, and the community, in order to deliver the highest quality of healthcare across settings.” For physicians and nurses, teamwork ability involves not only communication and social skills but also specific training on interdisciplinary work in order to be able to work together cooperatively, sharing responsibilities, solving problems, and making decisions to carry out actions centered on patients’ care (Hojat et al., 1999).

It has been reported that with the lack of any targeted interdisciplinary educational programs, important differences appear in the development of this ability between medicine and nursing (Onishi et al., 2013; Zheng et al., 2016; Tuirán-Gutiérrez et al., 2019). In addition, studies based on different training methodologies have demonstrated the important role that a targeted interdisciplinary educational program has in the development and improvement of this ability in medicine and nursing students (Raparla et al., 2017; Ferri et al., 2018; Tuirán-Gutiérrez et al., 2019). However, studies carried out in different cultural settings suggest that the cultural environment plays an important role of influence in an unbalanced development of this ability in healthcare professionals. Findings reported in Italy (Alcusky et al., 2016), Japan (Komi et al., 2011; Onishi et al., 2013), Singapore (Zheng et al., 2016), Mexico (Hojat et al., 2001), and Palestine (Elsous et al., 2017) indicate that nurses have a greater development of this ability in comparison with physicians, regardless of individual differences (such as personal or professional experiences). On the contrary, findings reported in the United States (Hojat et al., 1997, 2001, 2015; Hughes and Fitzpatrick, 2010) and in Israel (Hojat et al., 2003) were explained mainly by individual factors or factors associated with lack of training and professional experience. According to Veloski and Hojat (2006, p. 128), these cultural differences are associated with the fact that inter-professional relationships between medicine and nursing “tend to be hierarchical in societies where nurses have little autonomy and physicians dominate patient-care decisions. In contrast, inter-professional relationships tend to be ‘complementary’ in societies where physicians and nurses share power and where their roles and responsibilities are viewed as complementary.” Their assumption is based on the “principle of least interest,” initially proposed by Waller and Hill (1951) in family relationships, and on the “socialization role theory,” applied to physicians’ and nurses’ work relationships (Austin et al., 1985).

This issue acquires special relevance taking in consideration the important role that developing a “complementary” work relationship has in a healthcare institution. Its benefits are visible not only in patients’ care and clinical outcomes but also in core professional competences, such as: communication, innovation, creativity, decision-making, empathy, and lifelong learning abilities (Veloski and Hojat, 2006; Ceschi et al., 2014; Pedrazza et al., 2017; Tuirán-Gutiérrez et al., 2019). Furthermore, several studies have noticed the negative effects that a hierarchical work relationship has in nurses, such as: medical errors, burnout, cynicism, lack of autonomy, and motivation to work and learn (Muliira et al., 2012; Tang et al., 2013; Hakanen et al., 2014; Abdi et al., 2015; Hailu et al., 2016; San-Martín et al., 2017a).

In Latin America, the first study reporting the prevalence of a hierarchical work model between physicians and nurses was reported in Mexico (Hojat et al., 2001). Fifteen years later, two other studies evidenced that this attitudinal gap was present in other Latin American institutions (San-Martín et al., 2017a). More recently, another study in Mexico showed that this problem appears in the first year of undergraduate studies as a combined effect of the social environment in the university and the lack of a targeted interdisciplinary educational program (Tuirán-Gutiérrez et al., 2019). This educational gap is a pending task in Latin America, where an important part of the 6 years of medical training is done in public hospitals. Recent studies warn of a dominant hierarchical work culture in those public healthcare institutions (Rocha et al., 2014; San-Martín et al., 2017a; Rice et al., 2018). Finding out whether a long exposition to a hierarchical environment influences how medicine students develop their attitudes toward inter-professional collaboration with nurses is an important educational concern (Fox et al., 2018). Regarding this issue, a recent study performed in five Peruvian public hospitals showed that abilities toward inter-professional collaborative work did not improve in medicine students after 3 months of clinical training in those hospitals, as could be initially expected (San-Martín et al., 2016). These preliminary findings suggest that, in the absence of targeted interdisciplinary educational programs, the social environment acquires an important role of influence in the development of inter-professional collaborative work abilities. These findings are in accordance with the proposal suggested by Täuber (2018) that the social environment strongly influences human relationships and social interactions. Then, it is understandable that under a long exposition to a certain social environment, attitudes toward work relationships become stronger, regardless of other influencing factors associated with individual characteristics.

Some of these individual characteristics are related to social and emotional skills, which help individuals to interact cooperatively in group or to be more empathetic with others. Being empathetic implies having interpersonal and communication skills and the ability to understand others’ views, which facilitates a positive interaction with others (Greenberg et al., 2001). On the contrary, loneliness is defined as the perception that one lacks meaningful connections with others, indicating an absence of interpersonal skills (Hojat and Crandall, 1987) that is reflected in unsatisfactory human connections (DiTommaso and Spinner, 1997). Lonely individuals are likely to score low on measures of positive aspects of personality conducive to relationship building (Mellor et al., 2008). Lonely individuals are also less likely to trust others, suggesting that this experience is not conducive to forming empathetic relationships (Hojat, 1982). Such persons are therefore more prone to the self-fulfilling attributions that perpetuate loneliness, which in turn lead to less competent interactions (Spitzberg and Hunt, 1987). However, social and communication skills are not the only personal characteristics that could have an impact on the development of teamwork abilities. Lifelong learning and subjective well-being possibly play another role of influence. In the first case, lifelong learning is defined as “a concept involving a set of *self-initiated activities* (behavioral aspect) and *information-seeking skills* (capabilities) that are activated in individuals with a sustained *motivation* (predisposition) to learn and the ability to recognize their own learning needs” (Veloski and Hojat, 2006, p. 133). It is expected that individuals with high scores on lifelong learning measures could find in interdisciplinary environments an important opportunity for learning and improving their professional competences. In accordance with this, a positive association between lifelong learning and teamwork abilities has been reported in recent studies with physicians-in-training (San-Martín et al., 2017b) and with medical and nursing students (Tuirán-Gutiérrez et al., 2019). On the other hand, subjective well-being refers to individuals’ emotional and cognitive evaluation of their lives (VanderWeele et al., 2012). It is demonstrated that people with lower subjective well-being are socially withdrawn, leading to higher level of loneliness. On the contrary, people with higher subjective well-being act more positively toward others, prompting a positive response and close social ties (Fredrickson and Joiner, 2002). In summary, having empathetic orientation, motivation to learn, and high self-esteem, or on the contrary, perceiving barriers to having satisfactory human connections, could influence the development of satisfactory social interactions at the workplace.

Based on this research framework, this study was performed in order to test the following hypothesis: the development of abilities toward inter-professional collaborative work (teamwork) starts at very early stages of undergraduate studies and is sensitive to the main influence of *environmental factors*, associated with formal learning (targeted interdisciplinary educational programs) and with non-formal learning (dominant work relationship models). Other aspects (*individual factors*), mainly related to social and emotional skills oriented to the capacity that individuals have to interact cooperatively in group with others, have a secondary role in this matter. Then, in the absence of formal learning, teamwork ability in undergraduate students is mainly influenced by non-formal learning. Under those circumstances, having social and emotional skills is not sufficient to compensate the environmental effect, when it is contrary to a collaborative work model.

With this purpose, this study pursued three goals: (i) to measure the development of teamwork abilities in a sample of undergraduate students of medicine and nursing; (ii) to determine whether some of the following variables – gender, age, loneliness (as a negative indicator of relationship building skills), subjective well-being (measured by life satisfaction), and empathy and lifelong learning (two other specific components of professionalism described as complementary competences of teamwork abilities) – play a role of influence (as individual factors) in the development of teamwork abilities; and (iii) to determine whether, in the absence of specific programs of training in teamwork abilities (formal learning), some of the following variables associated with informal learning—academic achievement (measured by the number of semesters completed), discipline studied (medicine or nursing), and type of university where the students were enrolled (public or private) – play a role of influence (as environmental factors) in the development of teamwork abilities.

## Materials and Methods

### Participants

In the academic year 2018–2019, when this study was performed, the complete population of undergraduate students matriculated in the medical and nursing faculties of the three universities of Cusco city included 2,440 students (1,410 corresponded to two medical faculties and 1,030 to three nursing faculties). None of these five faculties offer in their curricula a specific course on inter-professional or interdisciplinary collaborative work abilities.

Due to geographical limitations of access, only undergraduate students attending academic activities in Cusco city were included. Those who were attending academic activities out of Cusco city by the time this study was performed, such as communitarian work in isolated rural communities and exchange and internship programs in other institutions, were excluded from the study. Students who complied with the inclusion criteria were invited to participate voluntarily and anonymously.

### Main Measures

To measure teamwork abilities, the Jefferson Scale of Attitudes toward Physician–Nurse Collaboration (JSAPNC) was used. The JSAPNC was developed in response to the need for a valid instrument to measure an important component of professionalism, namely, teamwork and inter-professional collaboration between physicians and nurses. According to the authors of the JSAPNC (Hojat et al., 1999), “physician–nurse collaboration is defined as an ability of nurses and physicians to work together cooperatively, sharing responsibilities for solving problems and making decisions to formulate and carry out plans for patient care.” The 15 items of the JSAPNC is answered using a Likert scale from 1 (*strongly disagree*) to 4 (*strongly agree*). This instrument has demonstrated a high reliability and validity among medical and nursing students in different cultural contexts (Ward et al., 2008; Hojat et al., 2015; Zakerimoghadam et al., 2015; Tuirán-Gutiérrez et al., 2019).

Two versions of the Jefferson Scale of Empathy (JSE) were applied to measure students’ orientation toward empathetic relationships with patients (Hojat, 2016): the medicine student version (JSE-S) for medicine students and the healthcare student version (JSE-HPS) for nursing students. The 20 items of the JSE are answered on a Likert scale from 1 (*strongly disagree*) to 7 (*strongly agree*). The difference between the two versions of the JSE used is in the rewording of those items where the terms “medicine” or “physician” appeared, in order to make it more applicable to students from other healthcare areas. A sample item of the JSE-S is: “Physicians should try to stand in their patients’ shoes when providing care to them,” while the same item in the JSE-HPS is reworded as follows: “Health care providers should try to stand in their patients’ shoes when providing care to them.” A higher score means that the student has a greater orientation or behavioral tendency toward empathic engagement in patient care (Hojat, 2016).

Two versions of the Jefferson Scale of Physician Lifelong Learning (JeffSPLL) were applied to measure attitudes toward lifelong learning: the medicine student version (JeffSPLL-MS), for medicine students (Wetzel et al., 2010) and the healthcare profession student version (JeffSPLL-HPS) for nursing students (Novak et al., 2014). The JeffSPLL was originally designed to measure the development of skills related to information gathering, the use of learning opportunities, and self-motivation (Hojat et al., 2009). The 14 items of the JeffSPLL are answered on a Likert scale from 1 (*strongly disagree*) to 4 (*strongly agree*). Similarly to the two versions of the empathy scale, the differences between the two versions of the JeffSPLL are in the rewording of those items where the term “medicine” is used, in order to make it more applicable to students from other healthcare areas. The JeffSPLL tool has demonstrated a high reliability and validity in both student groups in different cultural contexts (Muliira et al., 2012; San-Martín et al., 2016; Tuirán-Gutiérrez et al., 2019).

To measure loneliness perception, the short version of the Social and Emotional Loneliness Scale for Adults (SELSA-S) was used. The SELSA-S produces a global loneliness score, as well as scores for three dimensions of loneliness: “family,” “romantic,” and “social” (DiTommaso et al., 2004). The 15 items of the SELSA-S are answered on a Likert scale from 1 (*strongly disagree*) to 7 (*strongly agree*). Higher scores indicate a higher perception of loneliness. The following are sample items from each of the SELSA-S dimensions: “I do not feel satisfied with the friends that I have” and “I feel part of a group of friends” (social dimension); “I have a romantic partner to whose happiness I contribute” and “I have someone who fulfils my emotional needs” (romantic dimension); and “I feel alone when I’m with my family” and “I feel close to my family” (family dimension). The SELSA-S has demonstrated good psychometric properties: concurrent and discriminant validity (DiTommaso et al., 2004). In studies with healthcare professionals and students of nursing, the SELSA-S has shown a high reliability, with coefficients closer to 0.90 (Domínguez et al., 2017; Marilaf-Caro et al., 2017).

Subjective well-being refers to the emotional and cognitive self-perception of one’s personal life. In this study, the Satisfaction with Life Scale (SWLS) was used as measure of subjective well-being (Diener et al., 1985; VanderWeele et al., 2012). The SWLS is composed of five items that are answered using a Likert scale from 1 (*strongly disagree*) to 5 (*strongly agree*). A high score on the SWLS is associated with high life satisfaction and subjective well-being. The SWLS has good discriminant and convergent validity, demonstrated in different cultural contexts (VanderWeele et al., 2012; Marilaf-Caro et al., 2017).

### Complementary Form

Information regarding age, sex, discipline (medicine or nursing), academic achievement measured by semester enrolled (from 1 to 12 in the case of medicine studies and from 1 to 10 in the case of nursing studies), and type of university (public or private) was collected through a complementary form.

### Procedures

Questionnaires including the instruments and the complementary forms were administered to medicine and nursing students. The questionnaires consisted of paper forms provided together with an information letter in enclosed envelopes that were returned to the local researchers following a general protocol previously approved by an independent ethics committee (Ref. CEICLAR PI 199). All participant universities provided administrative support to the process of distribution and collection of the questionnaires. The work was carried out in accordance with the ethical principles for medical research involving human subjects of the Declaration of Helsinki. There was no potential risk for participants, and anonymity was guaranteed throughout the process.

### Statistical Assessment

Internal consistency reliability was calculated using Cronbach’s alpha coefficient. Following the guidelines suggested by the American Educational Research Association, values higher than 0.7 were considered satisfactory.

Once the normality of the inter-professional collaborative work scores was studied, a variance analysis (three-way ANOVA) based on discipline, sex, and university, as explanatory variables, was performed. Two-way interaction effects were also analyzed to determine if there were differences on inter-professional collaborative work measures defined by a combination of “sex by discipline,” “discipline by university,” and “sex by university.” Furthermore, a three-way interaction was also analyzed to determine if there were differences in scores of teamwork due to a combination among “discipline by sex and by university.” Finally, effect size was calculated using the eta-squared value in order to measure the ratio of variance explained in the dependent variable (teamwork) by each of the predictors studied while the others were controlled.

With the purpose of determining possible associations among inter-professional collaborative work abilities, two other components of professionalism (empathy and lifelong learning), academic achievement (measured by semester completed), loneliness, subjective well-being (measured by life satisfaction), and age, a correlation analysis using Spearman’s coefficient was performed.

All analyses were performed using R statistical software, version 3.5.1 for Windows. The statistical analyses of the data also included *multilevel* (Bliese, 2013) and *apaTable* (Stanley and Spence, 2018) packages.

## Results

The entire number of questionnaires distributed in the three universities of Cusco with medical and nursing faculties was 1,880. From them, 1,518 were answered and returned (response rate of 81%).

The study sample was composed of 817 (54%) medicine students (348 men and 469 women) and 700 (46%) nursing students (72 men and 628 women). The mean age of the participants was 22 years old, with a 17–57 years old age range (*SD* = 4.63). With regard to universities, 460 respondents were students enrolled in one public university (237 in the faculty of medicine and 223 in the faculty of nursing). The other 1,054 respondents were recruited in the other two universities (both private). From them, 801 students corresponded to one university (580 in the faculty of medicine and 221 in the faculty of nursing), and the other 253 were nursing students of the other private university. According to academic achievement (measured by semester enrolled), the range of semesters covered in the entire sample corresponded with the complete undergraduate programs of medicine (12 semesters) and nursing (10 semesters) that are offered by Peruvian medical and nursing schools, respectively. The five instruments used showed adequate psychometric properties measured by Cronbach’s coefficients higher than 0.70 in all cases. Since none of the five instruments used followed a normal distribution, non-parametric tests were performed in comparison and correlation analyses. The score distribution, descriptive statistics, and reliability for all instruments used in this study are described in Table 1.

**TABLE 1:** Descriptive statistics and psychometric reliability of scales of empathy, inter-professional collaboration, lifelong learning, loneliness, and life satisfaction (*n* = 1,518).

Statistics	JSAPNC	JSE	JeffSPLL	SELSA	SWLS
*n*	1,491	1,477	1,498	1,489	1,503
Possible range	15–60	20–140	14–56	15–105	5–25
Actual range	15–60	30–140	14–56	15–104	5–25
Mean	46	101	44	47	18
Standard deviation	9	19	7	15	5
**Percentile**					
25th	41	88	40	36	15
50th (median)	47	104	45	48	18
75th	53	115	49	58	21
Reliability	0.89	0.86	0.87	0.79	0.83

*JSAPNC, Jefferson Scale of Attitudes toward Physician–Nurse Collaboration; JSE, Jefferson Scale of Empathy; JeffSPLL, Jefferson Scale of Physician Lifelong Learning; SELSA, Social and Emotional Loneliness Scale for Adults; SWLS, Satisfaction with Life Scale.*

**FIGURE 1:**
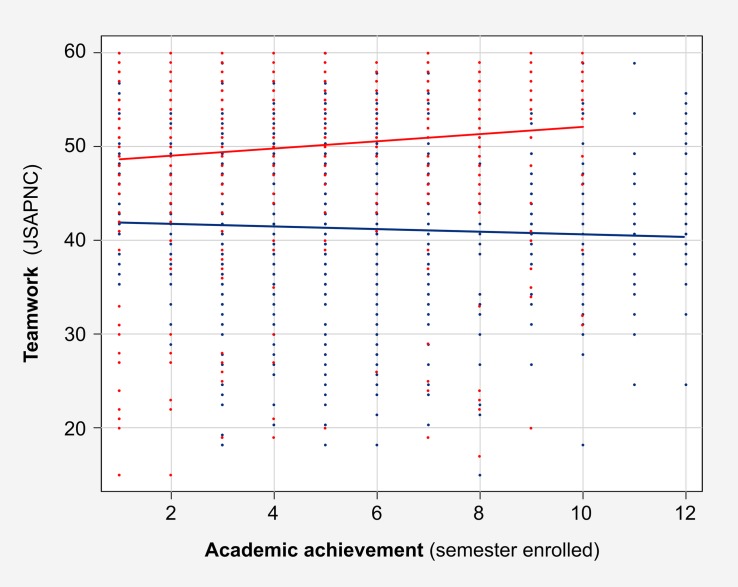
Global scores of the Jefferson Scale of Attitudes toward Physician–Nurse Collaboration (JSAPNC) in medicine students group (blue) and nursing students group (red) according to their academic achievement, measured by semester enrolled.

With regard to the first goal related to differences between teamwork abilities between medical and nursing students, no differences were found between the two private nursing schools when the global scores on the JSAPNC were compared (*p* = 0.65). Based on these preliminary findings, both groups were treated as a unique group in subsequent analyses. In the entire sample, the comparison analysis showed that nursing students had higher scores (*M* = 50; Mdn = 52; *SD* = 9) in teamwork abilities than medicine students (*M* = 43; Mdn = 43; *SD* = 7), and this difference was statically significant (*p* < 0.001). Those differences not only appeared in all academic courses that are comparable (from 1 to 10); they also increased in accordance with students’ academic achievement, as is shown in Figure 1.

Regarding the second and third goals, categorical variables (discipline, sex, and university) and numerical variables (scores of empathy, lifelong learning, loneliness, and life satisfaction; semesters completed; and age) were analyzed separately.

**FIGURE 2:**
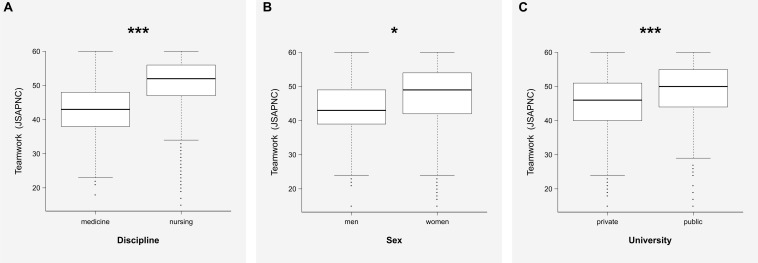
Comparisons of the global scores on the JSAPNC in the entire sample in relation to discipline **(A)**, sex **(B)**, and university **(C)**. *p = 0.01; ***p < 0.001.

In the group of categorical variables, a three-way ANOVA was performed showing differences according to discipline, sex, and university (Figure 2). A similar difference also appeared in the interaction among all of them: “discipline by sex and by university,” as is shown in Table 2. On the contrary, no differences appeared associated with the interaction of “sex by discipline” [*F*_(__1_, _1_,_478__)_ = 0.9; *p* = 0.34], “sex by university” [*F*_(__1_, _1_,_478__)_ = 1.8; *p* = 0.17], or “discipline by university” [*F*_(__1_, _1_,_478__)_ = 1.5; *p* = 0.22]. However, when the size effects of all variables were measured, the analysis showed that only discipline had a large effect in the variance of JSAPNC (η*_p_*^2^ = 0.14), while the other variables with statistical significance showed small effects in the variance of JSAPNC, as is shown in Table 2.

**TABLE 2:** Summary results of a three-way ANOVA of the scores of the inter-professional collaborative work by discipline, sex, and university (*n* = 1,518).

Source of variation	*F*(_1, 1,478_)	η^2^	η*_p_*^2^	*p*
**Main effects**				
Discipline (medicine vs. nursing)	247.9	0.13	0.14	<0.001
Sex (men vs. women)	6.4	0.003	0.004	0.01
University (public vs. private)	54.1	0.03	0.03	<0.001
**Three-way interaction**				
Discipline – sex – university	5.9	0.01	0.01	<0.001

*F, F value; η^2^, eta-squared; η_p_^2^, eta-partial-square; p, p-value.*

In the group of numerical variables, correlation analysis confirmed the existence of a positive association between empathy and teamwork abilities (ρ = +0.49; *p* < 0.001) and between teamwork and lifelong learning abilities (ρ = +0.48; *p* < 0.001). These associations appear in both groups: medical and nursing student groups. However, when the interaction between academic achievement and teamwork abilities was studied, important differences appeared according to discipline. While in the medicine students’ group, this association was inverse (ρ = −0.08; *p* = 0.02), in the nursing students’ group, this association was positive (ρ = +0.15; *p* < 0.001). With regard to loneliness, teamwork abilities showed an inverse correlation only with the social dimension of loneliness perception (ρ = −0.29; *p* < 0.001) and the family dimension of loneliness perception (ρ = −0.33; *p* < 0.001). On the contrary, no association was observed between teamwork abilities and the romantic dimension of loneliness perception, either in the entire sample (ρ = +0.03; *p* = 0.23) or in any of both student groups. On the other hand, subjective well-being (measured by life satisfaction) showed a positive correlation with teamwork abilities but only in the case of medicine students (ρ = +0.15; *p* < 0.001). Finally, no association between teamwork and age was observed, either in medical (ρ = −0.03; *p* = 0.41) or nursing student groups (ρ = −0.06; *p* = 0.11). A summary of all these analyses is shown in Table 3.

**TABLE 3:** Spearman’s correlation analysis among inter-professional collaborative abilities and measures of professionalism, academic achievements, loneliness, subjective well-being, and age (*n* = 1,518).

Variables	Entire sample		Medicine students		Nursing students	
	ρ	*p*	ρ	*p*	ρ	*p*
**Professionalism components**						
Empathy (JSE)	+ 0.49	<0.001	+ 0.45	<0.001	+ 0.59	<0.001
Lifelong learning (JeffSPLL)	+ 0.48	<0.001	+ 0.43	<0.001	+ 0.56	<0.001
**Academic achievement**						
Semester completed	–0.06	0.02	–0.08	0.02	+ 0.15	<0.001
**Loneliness**						
Global measure (SELSA-S)	–0.28	<0.001	–0.24	<0.001	–0.34	<0.001
Romantic dimension	+ 0.03	0.23	+ 0.04	0.25	+ 0.01	0.70
Social dimension	–0.29	<0.001	–0.26	<0.001	–0.34	<0.001
Family dimension	–0.33	<0.001	–0.27	<0.001	–0.38	<0.001
**Subjective well-being**						
Life satisfaction (SWLS)	+ 0.14	<0.001	+ 0.15	<0.001	+ 0.06	0.14
Age	+ 0.02	0.36	–0.03	0.41	–0.06	0.11

*SELSA-S, Social and Emotional Loneliness Scale for Adults, short version; ρ, Spearman’s coefficient. p, p-Value.*

## Discussion

The findings presented confirm the main hypothesis of this study that in the absence of a targeted interdisciplinary educational program, the development of teamwork abilities in undergraduate medical and nursing students can be limited, probably influenced by the socio-cultural environment. The factors of influence associated with the social environment depend on the professional role (to be a medical or a nursing student), the hierarchical culture (to be enrolled at the beginning or in advanced stages of undergraduate studies), and the elitist academic atmosphere (studying in a private or public university).

The outcomes observed in this study provide novel information regarding the attitudinal gap between medical and nursing students in Latin America, firstly reported in a recent study performed in Mexico (Tuirán-Gutiérrez et al., 2019). In this study, differences in teamwork abilities associated with discipline appear in a very early stage of undergraduate studies, similar to the one reported in Mexico. Furthermore, the novel information provided by this study indicates that those differences are bigger in advanced academic courses. In addition, differences associated with the type of university indicate that studying in a private university is related to a lower development of teamwork abilities. These three main findings are consistent with the negative effect that a hierarchical culture has in the development of teamwork abilities, previously reported in Latin American healthcare professionals (Hojat et al., 2001; Hojat et al., 2003; San-Martín et al., 2017a). The abovementioned outcomes provide new evidence supporting the important role that social environment plays in the enhancement of this attitudinal gap between medical and nursing students, which is in consonance with a theoretical model (Täuber, 2018), previously proved in other social contexts, that associates social environment and social behavior. This is also in consonance with the previously described “principle of least interest” and “socialization role theory” proposed by Waller and Hill (1951) and Austin et al. (1985), respectively.

But in addition to the environment, there are also individual factors that play a role of influence in the development of teamwork abilities. In this study, sex, loneliness, empathetic orientation, lifelong learning abilities, and subjective well-being showed a role of influence on the development of teamwork abilities. However, in comparison with the environment, this effect is smaller, as has been shown by the measurement of the size effect.

In the case of loneliness, the inverse correlation observed in both groups, medical and nursing students, corroborates that the lack of personal ability for establishing human connections is associated with the difficulty to establish adequate work relationships, measured by teamwork abilities. In this study, loneliness was measured in three domains: family, social network, and romantic relationships. Associations were found in the first two domains that specifically imply interaction with others, while in the third one, no association was observed.

In contrast to loneliness, the positive correlation observed between empathy and teamwork measures are in consonance with the conceptualization that interpersonal skills and understanding patients’ concerns are common denominators shared in empathetic engagement in patient care and in inter-professional collaborative care (Hojat et al., 2015). Findings indicate that being empathetic helps students to construct solid work relationships that are reflected in higher teamwork score measures, regardless of discipline. In the case of lifelong learning abilities, the positive correlation observed between these abilities and teamwork measures suggests that students with higher lifelong learning abilities find in interdisciplinary environments important possibilities to learn and improve their professional training. This finding is in accordance with the definition of lifelong learning as an attribute involving learning beliefs and motivations, attention to learning opportunities, and developing skills in seeking information (Wetzel et al., 2010).

Finally, findings observed in this study regarding subjective well-being, measured by life satisfaction, require especial attention. The outcomes observed in this study provide novel information regarding the role that subjective well-being plays in building collaborative work relationships. However, the difference observed between medical and nursing students also indicates that this effect could be mediated by the social environment. In a hierarchical work culture, medicine is associated with more managerial responsibilities, while nursing is associated with a subordinate role. This important difference in professional roles may drive medicine students with higher subjective well-being to be more motivated in acquiring abilities toward teamwork. Under the same circumstances, having higher scores in subjective well-being does not necessarily have similar effects in the development of nursing students’ teamwork abilities.

In conclusion, this study provides information confirming the predominant role of influence that the socio-cultural environment plays in the development of teamwork abilities in the absence of targeted educational programs. In societies where medicine is placed above nursing, this role of influence acquires more relevance. Furthermore, this influence can be reinforced in academic institutions where the focus of medical training is mainly placed on technical and clinical aspects rather than in communication and social work abilities, while nursing studies are preferably focused on care, communication, social skills, and collaborative work abilities. Although empathy and lifelong learning appear as two important competences that could help develop teamwork abilities, their influence is not strong enough to compensate for the negative effect of the environment and a dominant hierarchical work culture. These findings warn of the urgent need to include targeted educational programs on interdisciplinary collaborative work in different stages of undergraduate studies for the acquisition of this ability.

## Limitations

In this study, it was not possible to include students who were attending formative activities in small towns and rural communities of Cusco. These activities are performed (i) in settlements where social stereotypes associated with professional roles are possibly stronger and (ii) in circumstances when the accumulated effect of the exposition to a hierarchical culture is greater (advanced courses). Without including this minority group, important differences were observed linking this attitudinal gap in interdisciplinary collaborative work between medical and nursing students with the socio-cultural environment and a hierarchical culture. However, further studies in students enrolled in those activities could bring new information in this matter. This study was performed in five schools located in one Peruvian city in the Andean region. Even though Cusco has a relevant importance in the Andean region of Latin America due to its strategical position and multicultural and multilingual social structure, further studies in other geographical and cultural contexts are required in order to determine the existence of possible similarities.

## Data Availability Statement

The datasets generated for this study are available on request to the corresponding author.

## Ethics Statement

The studies involving human participants were reviewed and approved by Comité Ético de Investigación de La Rioja (CEICLAR). Written informed consent from the participants’ legal guardian/next of kin was not required to participate in this study in accordance with the national legislation and the institutional requirements.

## Author Contributions

LV was in charge of the study’s overall design and drafting of the manuscript. MS-M and LV performed the statistical processing of data. NB-T, LC-A, SC, and EG were in charge of coordination with the participating institutions. BC-C and PM were in charge of surveys design, distribution, and data collection. LV and RD prepared manuscript drafts. All authors contributed to the presented work, participated during the interpretation process of the results, and approved the final manuscript.

## Conflict of Interest

The authors declare that the research was conducted in the absence of any commercial or financial relationships that could be construed as a potential conflict of interest.

## References

[B1] Abdi Z. Delgoshaei B. Ravaghi H. Abbasi M. Heyrani A. (2015). The culture of patient safety in an Iranian intensive care unit. *J. Nurs. Manag.* 23 333–345. 10.1111/jonm.12135 23902287

[B2] Alcusky M. Ferrari L. Rossi G. Liu M. Hojat M. Maio V. (2016). Attitudes toward collaboration among practitioners in newly established medical homes: a survey of nurses, general practitioners, and specialists. *Am. J. Med. Qual.* 31 526–535. 10.1177/1062860615597744 26228578

[B3] Austin J. K. Champion V. L. Tzeng O. C. S. (1985). Crosscultural comparison on nursing image. *Int. J. Nurs. Stud.* 22 231–239. 10.1016/0020-7489(85)90006-90009 3850079

[B4] Bliese P. (2013). *Multilevel Functions. R Package Version 2.5.*

[B5] Ceschi A. Dorofeeva K. Sartori R. (2014). Studying teamwork and team climate by using a business simulation: how communication and innovation can improve group learning and decision-making performance. *Eur. J. Train. Dev.* 38 211–230. 10.1108/ejtd-01-2013-0004

[B6] Diener E. Emmons R. A. Larsen R. J. Griffin S. (1985). The satisfaction with life scale. *J. Pers. Assess.* 49 71–75. 10.1207/s15327752jpa4901_13 16367493

[B7] DiTommaso E. Brannen C. Best L. A. (2004). Measurement and validity characteristics of the short version of the social and emotional loneliness scale for adults. *Educ. Psychol. Meas.* 64 99–119. 10.1177/0013164403258450 31349835

[B8] DiTommaso E. Spinner B. (1997). Social and emotional loneliness: a re-examination of Weiss’ typology of loneliness. *Pers. Individ. Differ.* 22 417–427. 10.1016/S0191-8869(96)00204-8 6737214

[B9] Domínguez V. San-Martín M. Vivanco L. (2017). Family relationships, loneliness, and empathy in patient care in student nurses. *Aten. Primaria* 49 56–57. 10.1016/j.aprim.2016.03.007 27350409 PMC6876022

[B10] Elsous A. Radwan M. Mohsen S. (2017). Nurses and physicians attitudes toward nurse-physician collaboration: a survey from Gaza Strip, Palestine. *Nurs. Res. Pract.* 2017:7406278. 10.1155/2017/7406278 28326194 PMC5343283

[B11] Ferri P. Rovesti S. Magnani D. Barbieri A. Bargellini A. Mongelli F. (2018). The efficacy of interprofessional simulation in improving collaborative attitude between nursing students and residents in medicine. a study protocol for a randomised controlled trial. *Acta Biomed.* 89 32–40. 10.23750/abm.v89i7-S.7875 30539929 PMC6502140

[B12] Fox L. Onders R. Hermansen-Kobulnicky C. J. Nguyen T. N. Myran L. Linn B. (2018). Teaching interprofessional teamwork skills to health professional students: a scoping review. *J. Interprof. Care.* 32 127–135. 10.1080/13561820.2017.1399868 29172791

[B13] Fredrickson B. L. Joiner T. (2002). Positive emotions trigger upward spirals toward emotional well-being. *Psychol. Sci.* 13 172–175. 10.1111/1467-9280.00431 11934003

[B14] Greenberg L. S. Watson J. C. Elliot R. Bohart A. C. (2001). Empathy. *Psychotherapy* 38 380–384. 10.1037/0033-3204.38.4.380

[B15] Hailu F. B. Kassahun C. W. Kerie M. W. (2016). Perceived nurse-physician communication in patient care and associated factors in public hospitals of jimma zone, South West Ethiopia: cross sectional study. *PLoS One* 11:e0162264. 10.1371/journal.pone.0162264 27632162 PMC5025155

[B16] Hakanen J. J. Perhoniemi R. Bakker A. B. (2014). Crossover of exhaustion between dentists and dental nurses. *Stress Health* 30 110–121. 10.1002/smi.2498 23723149

[B17] Hojat M. (1982). Loneliness as a function of selected personality variables. *J. Clin. Psychol.* 38 137–141. 10.1002/1097-4679(198201)38:1<137::aid-jclp2270380122>3.0.co;2-2 7056862

[B18] Hojat M. (2016). *Empathy in Health Professions Education and Patient Care.* Cham: Springer.

[B19] Hojat M. Bianco J. A. Mann D. Massello D. Calabrese L. H. (2015). Overlap between empathy, teamwork and integrative approach to patient care. *Med. Teach.* 37 755–758. 10.3109/0142159X.2014.971722 25314019

[B20] Hojat M. Crandall R. (1987). Loneliness: theory, research, and applications. *J. Soc. Behav. Pers.* 2 271–272.

[B21] Hojat M. Fields S. K. Rattner S. L. Griffiths M. Cohen M. J. Plumb J. D. (1997). Attitudes toward physician-nurse alliance: comparisons of medical and nursing students. *Acad. Med.* 72 S1–S3. 10.1097/00001888-199710001-00001 9347721

[B22] Hojat M. Fields S. K. Veloski J. J. Griffiths M. Cohen M. J. Plumb J. D. (1999). Psychometric properties of an attitude scale measuring physician-nurse collaboration. *Eval. Health Prof.* 22 208–220. 10.1177/01632789922034275 10557856

[B23] Hojat M. Gonnella J. S. Nasca T. J. Fields S. K. Cicchetti A. Lo Scalzo A. (2003). Comparisons of American, Israeli, Italian and Mexican physicians and nurses on the total and factor scores of the Jefferson scale of attitudes toward physician-nurse collaborative relationships. *Int. J. Nurs. Stud.* 40 427–435. 10.1016/S0020-7489(02)00108-6 12667519

[B24] Hojat M. Nasca T. J. Cohen M. J. Fields S. K. Rattner S. L. Griffiths M. (2001). Attitudes toward physician-nurse collaboration: a cross-cultural study of male and female physicians and nurses in the United States and Mexico. *Nurs. Res.* 50 123–128. 10.1097/00006199-200103000-00008 11302292

[B25] Hojat M. Veloski J. J. Gonnella J. S. (2009). Measurement and correlates of physicians’ lifelong learning. *Acad. Med.* 84 1066–1074. 10.1097/ACM.0b013e3181acf25f 19638773

[B26] Hughes B. Fitzpatrick J. J. (2010). Nurse-physician collaboration in an acute care community hospital. *J. Interprof. Care* 24 625–632. 10.3109/13561820903550804 20807034

[B27] Komi K. Onishi M. Kanda K. (2011). Development of the Japanese version of the jefferson scale of attitudes toward physician–nurse collaboration and measuring physicians’ and nurses’ attitudes toward collaboration in Japan. *Med. Educ. Jpn.* 42 9–17. 10.11307/mededjapan.42.9

[B28] Marilaf-Caro M. San-Martín M. Delgado-Bolton R. Vivanco L. (2017). Empathy, loneliness, burnout, and life satisfaction in Chilean nurses of palliative care and homecare services. *Enferm. Clin.* 27 379–386. 10.1016/j.enfcli.2017.04.007 28587755

[B29] Mellor D. Stokes M. Firth L. Hayashi Y. Cummins R. (2008). Need for belonging, relationship satisfaction, loneliness, and life satisfaction. *Pers. Individ. Differ.* 45 213–218. 10.1016/j.paid.2008.03.020

[B30] Muliira J. K. Etyang C. Muliira R. S. Kizza I. B. (2012). Nurses’ orientation toward lifelong learning: a case study of Uganda’s National Hospital. *J. Contin. Educ. Nurs.* 43 90–96. 10.3928/00220124-20111003-03 21985072

[B31] Novak M. K. Palladino C. Ange B. Richardson D. (2014). Measuring health professions students’ orientation toward lifelong learning. *J. Allied Health* 43 146–149. 25194060

[B32] Onishi M. Komi K. Kanda K. (2013). Physicians’ perceptions of physician–nurse collaboration in Japan: effects of collaborative experience. *J. Interprof. Care.* 27 231–237. 10.3109/13561820.2012.736095 23134378

[B33] Pedrazza M. Sartori R. Berlanda S. (2017). “Inter-professional collaboration: an evaluation study,” in *Innovating in Practice*, eds Russo-Spena T. Mele C. Nuutinen N. (Cham: Springer), 487–507. 10.1007/978-3-319-43380-6_21

[B34] Raparla N. Davis D. Shumaker D. Kumar A. Hafiz S. Sava J. (2017). A pilot program to improve nursing and surgical intern collaboration: lessons learned from a mixed-methods study. *Am. J. Surg.* 213 292–298. 10.3109/13561820.2012.736095 28017298

[B35] Rice H. E. Lou-Meda R. Saxton A. T. Johnston B. E. Ramirez C. C. Mendez S. (2018). Building a safety culture in global health: lessons from Guatemala. *BMJ Glob. Health* 3:e000630. 10.1136/bmjgh-2017-000630 29607099 PMC5873535

[B36] Rocha F. L. Marziale M. H. de Carvalho M. C. Cardeal Id. Sde F. de Campos M. C. (2014). The organizational culture of a Brazilian public hospital. *Rev. Esc. Enferm. U.S.P.* 48 308–314. 10.1590/S0080-6234201400002000016 24918891

[B37] San-Martín M. Delgado-Bolton R. Vivanco L. (2017a). Professionalism and occupational well-being: similarities and differences among latin american health professionals. *Front. Psychol.* 8:63. 10.3389/fpsyg.2017.00063 28179893 PMC5263132

[B38] San-Martín M. Rivera E. M. Alcorta-Garza A. Vivanco L. (2016). Moral perception, educational environment, and development of medical professionalism in medical students during the clinical rotations in Peru. *Int. J. Ethics Educ.* 1 163–172. 10.1007/s40889-016-0017-8

[B39] San-Martín M. Roig-Carrera H. Villalonga-Vadell R. M. Benito-Sevillano C. Torres-Salinas M. Claret-Teruel G. (2017b). Empatía, habilidades de colaboración interprofesional y aprendizaje médico permanente en residentes españoles y latinoamericanos que inician los programas de formación médica especializada en España. Resultados preliminares. *Aten. Prim.* 49 6–12. 10.1016/j.aprim.2016.02.007 27137344 PMC6876012

[B40] Spitzberg B. H. Hunt H. T. (1987). The relationship of interpersonal competence and skills to reported loneliness across time. *J. Soc. Behav. Pers.* 2 157–172.

[B41] Stanley D. Spence J. R. (2018). *Create American Psychological Association (APA) Style Tables, version 2.0.5 Cran R Project.*

[B42] Stern D. T. (2006). “A framework for measuring professionalism,” in *Measuring Medical Professionalism*, ed. Stern D. (New York, NY: Oxford University Press), 3–14.

[B43] Tang C. J. Chan S. W. Zhou W. T. Liaw S. Y. (2013). Collaboration between hospital physicians and nurses: an integrated literature review. *Int. Nurs. Rev.* 60 291–302. 10.1111/inr.12034 23961790

[B44] Täuber S. (2018). Moralized health-related persuasion undermines social cohesion. *Front. Psychol.* 9:909. 10.3389/fpsyg.2018.00909 29946279 PMC6005884

[B45] Tuirán-Gutiérrez G. J. San-Martín M. Delgado-Bolton R. Bartolomé B. Vivanco L. (2019). Improvement of inter-professional collaborative work abilities in mexican medical and nursing students: a longitudinal study. *Front. Psychol.* 10:5. 10.3389/fpsyg.2019.00005 30697172 PMC6340986

[B46] VanderWeele T. J. Hawkley L. C. Cacioppo J. T. (2012). On the reciprocal association between loneliness and subjective well-being. *Am. J. Epidemiol.* 176 777–784. 10.1093/aje/kws173 23077285 PMC3571255

[B47] Veloski J. Hojat M. (2006). “Measuring specific elements of professionalism: empathy, teamwork, and lifelong learning,” in *Measuring Medical and Professsionalism*, ed. Stern D.T. (New York, NY: Oxford University Press), 117–146.

[B48] Vivanco L. Delgado-Bolton R. (2015). ““Professionalism”,” in *Encyclopedia of Global Bioethics*, ed. Ten Have H. (Cham: Springer).

[B49] Waller W. Hill R. (1951). *The Family: A Dynamic Interpretation.* Ft Worth: Dryden Press.

[B50] Ward J. Schaal M. Sullivan J. Bowen M. E. Erdmann J. B. Hojat M. (2008). The Jefferson scale of attitudes toward physician–nurse collaboration: a study with undergraduate nursing students. *J. Interprof. Care* 22 375–386. 10.1080/13561820802190533 18800279

[B51] Wetzel A. P. Mazmanian P. E. Hojat M. Kreutzer K. O. Carrico R. J. Carr C. (2010). Measuring medical students’ orientation toward lifelong learning: a psychometric evaluation. *Acad. Med.* 85 S41–S44. 10.1097/ACM.0b013e3181ed1ae9 20881701

[B52] World Health Organization [WHO] (2010). *Framework for Action on Inter-Professional Education & Collaborative Practice.* Geneva: World Health Organization.

[B53] Zakerimoghadam M. Ghiyasvandian S. Kazemnejad-Leili A. (2015). Nurse–physician collaboration: the attitudes of baccalaureate nursing students at Tehran University of medical sciences. *Iran. Red Crescent Med. J.* 17:e23247 10.5812/ircmj.17(4)2015.23247 PMC4443301 26023338

[B54] Zheng R. M. Sim Y. F. Koh G. C. H. (2016). Attitudes towards interprofessional collaboration among primary care physicians and nurses in Singapore. *J. Interprof. Care* 30 505–511. 10.3109/13561820.2016.1160039 27269233

